# Mobile genetic elements drive the multidrug resistance and spread of *Salmonella* serotypes along a poultry meat production line

**DOI:** 10.3389/fmicb.2023.1072793

**Published:** 2023-03-16

**Authors:** Gabriel I. Krüger, Coral Pardo-Esté, Phillippi Zepeda, Jorge Olivares-Pacheco, Nicolas Galleguillos, Marcia Suarez, Juan Castro-Severyn, Luis Alvarez-Thon, Mario Tello, Jorge H. Valdes, Claudia P. Saavedra

**Affiliations:** ^1^Laboratorio de Microbiología Molecular, Facultad de Ciencias de la Vida, Universidad Andres Bello, Santiago, Chile; ^2^Grupo de Resistencia Antibacteriana en Bacterias Patógenas Ambientales GRABPA, Instituto de Biología, Pontificia Universidad Católica de Valparaíso, Valparaíso, Chile; ^3^Laboratorio de Microbiología Aplicada y Extremófilos, Departamento de Ingeniería Química, Universidad Católica del Norte, Antofagasta, Chile; ^4^Facultad de Ingeniería y Arquitectura, Universidad Central de Chile, Santiago, Chile; ^5^Laboratorio de Metagenómica Bacteriana, Centro de Biotecnología Acuícola, Departamento de Biología, Facultad de Química y Biología, Universidad de Santiago de Chile, Santiago, Chile; ^6^Center for Bioinformatics and Integrative Biology, Facultad de Ciencias de la Vida, Universidad Andres Bello, Santiago, Chile

**Keywords:** *Salmonella*, genomics, poultry, antimicrobial resistance, mobile genetics elements

## Abstract

The presence of mobile genetic elements in *Salmonella* isolated from a chicken farm constitutes a potential risk for the appearance of emerging bacteria present in the food industry. These elements contribute to increased pathogenicity and antimicrobial resistance through genes that are related to the formation of biofilms and resistance genes contained in plasmids, integrons, and transposons. One hundred and thirty-three *Salmonella* isolates from different stages of the production line, such as feed manufacturing, hatchery, broiler farm, poultry farm, and slaughterhouse, were identified, serotyped and sequenced. The most predominant serotype was *Salmonella* Infantis. Phylogenetic analyses demonstrated that the diversity and spread of strains in the pipeline are serotype-independent, and that isolates belonging to the same serotype are very closely related genetically. On the other hand, *Salmonella* Infantis isolates carried the pESI IncFIB plasmid harboring a wide variety of resistance genes, all linked to mobile genetic elements, and among carriers of these plasmids, the antibiograms showed differences in resistance profiles and this linked to a variety in plasmid structure, similarly observed in the diversity of *Salmonella* Heidelberg isolates carrying the IncI1-Iα plasmid. Mobile genetic elements encoding resistance and virulence genes also contributed to the differences in gene content. Antibiotic resistance genotypes were matched closely by the resistance phenotypes, with high frequency of tetracycline, aminoglycosides, and cephalosporins resistance. In conclusion, the contamination in the poultry industry is described throughout the entire production line, with mobile genetic elements leading to multi-drug resistant bacteria, thus promoting survival when challenged with various antimicrobial compounds.

## 1. Introduction

Food-borne diseases are taken as a main biological concern in the food industry and public health, and *Salmonella enterica* as one of the most common etiological agents (Wotzka et al., 2017). The disease caused by *Salmonella* contamination is one of the most recurrent worldwide zoonosis originated from food (Majowicz et al., 2010; EFSA, 2018; Fazza et al., 2021; Lee and Yoon, 2021). The current demand for food, as well as the production practices themselves, such as the overcrowding of cages, creates several risks of contamination, which leads to the increasing appearance and persistence of pathogens in the food industry. The main reservoir of *Salmonella* is the gastrointestinal tract of the host, yet the resulting contamination is able to spread and remain on surfaces throughout production (Golden et al., 2021).

Several serotypes of *S. enterica* have been reported in the poultry industry, with traits of concern for food safety (Oscar, 2021; O’Bryan et al., 2022), namely profiles of multi-drug resistance (MDR) and the increased prevalence of virulent serotypes in farm animals and humans (Shah et al., 2017).

Significant non-typhoid serotypes are Infantis (Mughini-Gras et al., 2021; Pardo-Esté et al., 2021), Typhimurium, Enteritidis (Karabasanavar et al., 2020), and Heidelberg (Dominguez et al., 2021), among others (Sun et al., 2021). The main cause of multi-resistant capacity is the indiscriminate use of antimicrobials, and the intensive use of cleaning and sterilization processes that are selective pressures upon the strains (Mahnert et al., 2015; Obe et al., 2021). These MDR strains are commonly found in poultry farms around the world, including Chile (Castro-Vargas et al., 2020; Pardo-Esté et al., 2021). In this context, the serotype Infantis is a worldwide-emerging serotype that is classified as one of the most prevalent non-typhoidal *Salmonella* in humans in Europe (EFSA, 2019). Furthermore, several reports indicate that the serotype Infantis is the most prevalent one in the poultry industry (EFSA, 2019; Vinueza-Burgos et al., 2019).

Diverse molecular mechanisms in non-typhoid *Salmonella* favor survival under various types of stresses (Whitehead et al., 2011; Kim et al., 2022). Such conditions, like the use of disinfection protocols or antimicrobial agents, are found in an industrial setting and can trigger a stress response. However, excessive or indiscriminate disinfection induces tolerance to these agents (Ortega Morente et al., 2013), causing the appearance of strains that are potentially resistant to antimicrobial agents along the production line, which can generate a worrying epidemiological scenario in which MDR strains can emerge.

Given this potential epidemiological risk, the rapid identification of Enterobacteriaceae strains, typing and molecular characterization of *Salmonella* using whole-genome sequencing to identify genomic profiles of interest to the poultry industry, has become a necessity (Park et al., 2014; Gymoese et al., 2019; Pardo-Esté et al., 2021). These constitute useful tools for the genomic surveillance of specific strains in outbreaks related to the industry or in epidemiological research. By typifying the whole-genome, genetic “fingerprints” that are specific for each site and time of isolation can be generated in order to further evaluate the epidemiology of an outbreak based on variances and mutations. These characterizations of *Salmonella* genomes isolated from the production line have described genetic determinants that confer resistance and virulence (de Melo et al., 2021; Mohamed et al., 2021; Zakaria et al., 2022). It is important to highlight that many of these determinants are present in mobile elements such as plasmids (Aviv et al., 2014; Kürekci et al., 2021; Tyson et al., 2021), integrons (Badouei et al., 2021), and transposons (Galetti et al., 2021). It is these genetic elements that cause a high risk of spreading antimicrobial resistance through horizontal gene transfer. Recently, an increase in the prevalence of *Salmonella* with an MDR profile has been described in the poultry industry (Gambino et al., 2022; Pławińska-Czarnak et al., 2022).

In this context, mobile genetic elements are involved in the ability of the bacteria to adapt to stress pressures (Hull et al., 2022). Despite this, the relationship of *Salmonella* and these elements within the production line environment of a chicken farm remains understudied. An understanding of these molecular factors would contribute to the comprehension and mitigation of widespread contamination. Therefore, in this study, we analyzed *Salmonella* populations isolated from a poultry farm in Chile, characterized them, and determined their genetic profiles, focusing on the presence of mobile genetic elements that contribute to pathogenicity and antimicrobial resistance.

## 2. Materials and methods

### 2.1. Study design

In this study, we characterized and compared 133 genomes of *S. enterica* isolates obtained from a production line in a poultry farm in 2018–2021, 30 isolates previously characterized (Pardo-Esté et al., 2021) and 103 isolates characterized in this work. Strains were isolated from the feed, hatchery, broiler, poultry farm, and slaughterhouse. Sampling, *Salmonella* isolation, and corroboration were performed as previously described (Pardo-Esté et al., 2021). All isolates were serotyped by Check & Trace (Check-Points BC™, Netherlands). The distribution of the isolates is detailed in Table 1 (for more details see Supplementary Table S1).

**Table 1:** Distribution of the isolated serotypes in the production line.

Serotype	Feed manufacturing	Hatchery	Broiler farm	Slaughterhouse	Poultry farm	Total
Agona	5	0	1	9	4	19
Corvallis	1	9	1	6	9	26
Heidelberg	10	2	2	15	20	16
Infantis	0	3	0	3	10	49
Senftenberg	14	4	0	3	2	23
Total	30	18	4	36	45	133

### 2.2. Antibiogram

All sequenced isolated were tested against a panel of 20 antibiotics using the disk diffusion method following CLSI guidelines (CLSI, 2018) for Enterobacter ales bacteria group. Antibiotic tested included: ampicillin (AMP, 10 μg); cefazolin (KZ, 30 μg); cefepime (FEP, 30 μg); ceftazidime (CAZ, 30 μg); ceftriaxone (CRO, 30 μg); ciprofloxacin (CIP, 5 μg); gentamicin (GEN, 10 μg); amikacin (AMK, 30 μg); imipenem (IPM, 10 μg); meropenem (MEM, 10 μg); ertapenem (ETP, 10 μg); tetracycline (TCY, 30 μg); ceftazidime/avibactam (CZA, 10/4 μg); piperacillin/tazobactam (TZP, 100/10 μg); trimethoprim/sulfamethoxazole (SXT, 1.25/23.75 μg); ampicillin/sulbactam (SAM, 10/10 μg); nitrofurantoin (NIT, 200 μg); chloramphenicol (CHL, 30 μg); and aztreonam (ATM, 30 μg), all of which were supplied by OXOID (Hampshire, England). Isolates resistant to three or more antimicrobial classes were cataloged as MDR.

### 2.3. DNA extraction and whole-genome sequencing of 133 strains

To extract the strain genomic DNA material, we used a commercial kit (Quick-DNA Miniprep Kit, Zymo Research) following the manufacturer’s instructions. The amount and quality of the extracted DNA was evaluated by fluorometry (Qubit 3.0, Thermo Fisher Scientific), while integrity was confirmed by capillary electrophoresis (LabChip GX Touch Nucleic Acid Analyzer, PerkinElmer, Spain). The DNA samples were sent to MIGS Center (Pittburgh, PA, USA) for paired-end library construction (2 × 151 bp paired-end reads) and sequenced in the NextSeq 2000 platform (Illumina Inc., San Diego, CA, USA). We used FastQC v0.11.9 (Andrews, 2010) for quality control and Trim-Galore v0.6.6 (Krueger, 2012) for filtering and trimming (-quality 30-trim-n-retain_unparied). Moreover, we used SPAdes v3.15.2 (Bankevich et al., 2012) for genome assembly (−isolate −k 33,55,77,99,111). The quality of the contigs was evaluated using QUAST v5.0.2 (Gurevich et al., 2013) and Depth coverage was determined by assigning the reads to the assemblies using Bowtie2 v2.4.2 (Langmead and Salzberg, 2012) and Samtools v1.12 (Danecek et al., 2021). The coding sequence predictions for genes and functional annotation were carried out with Prokka v1.14.6 (Seemann, 2014) and eggNOG-mapper v2.1.01 (Huerta-Cepas et al., 2017) using the EggNOG v5.0.2 (Huerta-Cepas et al., 2019). The completeness of the assembly was evaluated by identifying the ortholog markers for specific lineages, using BUSCO v5.2.2 (Manni et al., 2021) and checkM v1.1.3 (Parks et al., 2015). The genome assemblies generated in this research have been deposited at the DDBJ/ENA/GenBank under the Bioproject: PRJNA890630.

### 2.4. Bioinformatics analyses

#### 2.4.1. Genoserotyping and MLST analysis *in silico* typification and serotypification

Serotype predictions were performed by SeqSero2 v1.2.1 (Zhang et al., 2019) using the assembled genomes (−t 4 −k a). The information from the reads (−t 2 −m k) was corroborated by identifying in the data base the serotype determinants for the *Salmonella* genus (cluster *rfb, fliC* y *fljB*). Also, using the PubMLST data base for *S. enterica* (senterica) (Jolley et al., 2018), housekeeping genes *aroC*, *dnaN*, *hemD*, *hisD*, *purE*, *sucA*, and *thrA* were evaluated using mlst v1.2.1 (Page et al., 2016).

#### 2.4.2. Core genome Single Nucleotide Polymorphism (SNP) analysis and phylogeny

To determine all the genetic markers present in all the genomes from the isolates, we use GET_PHYLOMARKER v2.3.1 (Vinuesa et al., 2018) with the default parameters together with the GET_HOMOLOGUES (Contreras-Moreira et al., 2017) data output performed with the default parameters for the 133 annotated genomes. Additionally, using SNPs-sites v2.5.1 (Page et al., 2016), we determined the SNPs in the exit alignment (−c as exit). Then, the phylogeny was generated with IQ-TREEv2.2.0-beta (Nguyen et al., 2015), using the model for substitution GTR+ ABS, with 10,000 Bootstraps and selecting the best tree every 1,000 iterations. The results were visualized using FigTree v1.4.4 including the origin and the serotype of each strain. Also, from the SNP sequences from SNPs-site, we calculated the pairwise distance matrix between the genomes using snp-dists 0.8.2., which were then visualized with the Seaborn v0.11.2 package.

#### 2.4.3. Plasmid replicon detection and generation of draft plasmid sequences

We used PlasmidFinder v2.1 in default setting with the following parameters for threshold and coverage: 95% identity, 100% coverage, and 1×10^−5^ e-value, to identify plasmid replicons in genomes. We used the PLSDB database (Galata et al., 2019) to filter and create a specific database for each identified replicon. Then, using BLAST 2.12.0 (Altschul et al., 1990), we compared the contigs containing the identified replicon with the database created to select a reference plasmid, using the following parameters as selection criteria: >75% identity, >75% coverage and 1 × 10^−5^ e value. The first hit was chosen as the reference plasmid. Using BLAST, the contigs of the genome with more than 10 kbp were aligned with the reference plasmid and those that met the selection criteria were selected as part of the plasmid: >75% identity, >50% coverage and 1 × 10^−5^ e value. We chose an identity threshold of 75% because the replicon and the genes that make up the plasmid may be present in other bacteria and may not be conserved. On the other hand, 50% coverage was used to include discontinuous contigs, thus allowing the identification of breaks in the continuity of the plasmid due to other mobile elements, insertions or deletions. Finally, the smallest contigs (>3,000—<10,000) were aligned with the reference plasmid and evaluated by BLAST with the database created. If such hits met the criteria described above, these contigs were selected and denoted as part of the same plasmid. Selected contigs were extracted from the genome and used to generate a draft plasmid. Synteny was then ordered with the reference plasmid and saved for further analysis in fasta format. The isolates presenting the replicon of the Col plasmids were not processed in drafts due to the short sequences of the contigs and the difficulty of their assembly. However, contigs harboring the replicon and an antimicrobial resistance gene in the same sequence were analyzed as plasmids corresponding to the replicon.

#### 2.4.4. Detection of insertion sequences, transposons and integrons

The identification of other mobile elements in the draft plasmids, such as transposases and integrases (as well as their integration sequence), was carried out by BLAST using the TnCentral (Ross et al., 2021), ISFinder (Siguier et al., 2006), and Integrall (Moura et al., 2009) databases. The cut-off thresholds were 95% identity, 100% coverage, and a 1×10^−5^ e-value.

#### 2.4.5. Detection of virulence and resistance genes

We used the Comprehensive Antibiotic Resistance Database (CARD) to identify resistance elements (Alcock et al., 2019) to antibiotics and microbicides in the assembled genomes and the draft plasmids. Also, we used Virulence Factor Database (VFDB) to identify virulence factors. The thresholds to identify the virulence and resistance factors were 95% identity, 100% coverage, and a 1 × 10^−5^ *e*-value.

### 2.5. Statistical analyses

Antimicrobial susceptibility data were tabulated in csv format. Intermediate resistance collapsed into the susceptible category when resistance was represented as a binary variable. The presence or absence of a known resistance gene was compared with the interpretation of resistant or susceptible phenotypes when cultivated on the corresponding antimicrobial agent. Agreement measurements between phenotypic and genotypic results were performed using Cohen’s Kappa statistic (κ). Where the strength of agreement Cohen’s Kappa coefficient ranges from 0 to 0.2 none to slight agreement, 0.2–0.4 fair agreement, 0.4–0.6 moderate agreement, 0.6–0.8 good agreement, and 0.8–1.0 very good agreement.

## 3. Results

### 3.1. *In silico* serotypification of the *Salmonella* isolates

Overall, 133 isolates were serotyped using the microarray-based method Check&Trace (Check-Points, The Netherlands), from which the strains were classified into five serotypes: Agona, Corvallis, Heidelberg, Infantis, and Senftenberg. On the other hand, the SeqSero tool enabled us to perform analyses of genoserotypes from the WGS data for the 133 isolates coinciding with the results obtained by Check&Trace. From these analyses, we also identified 5 serotypes: 36.8% (49/133) of the isolates correspond to Infantis, 18.8% (25/133) Corvallis, 14.3% (19/133) Heidelberg, 14.3% (19/133) Agona, and 12.8% (17/133) Senftenberg. In addition, typing of the genome by MultiLocus Sequence Typing (MLST) described at least one Sequence Type (ST) strain typing for each serotype, of which the Senftenberg serotype had two types of strains.

Phylogeny of the *Salmonella* isolates.

**Figure 1:**
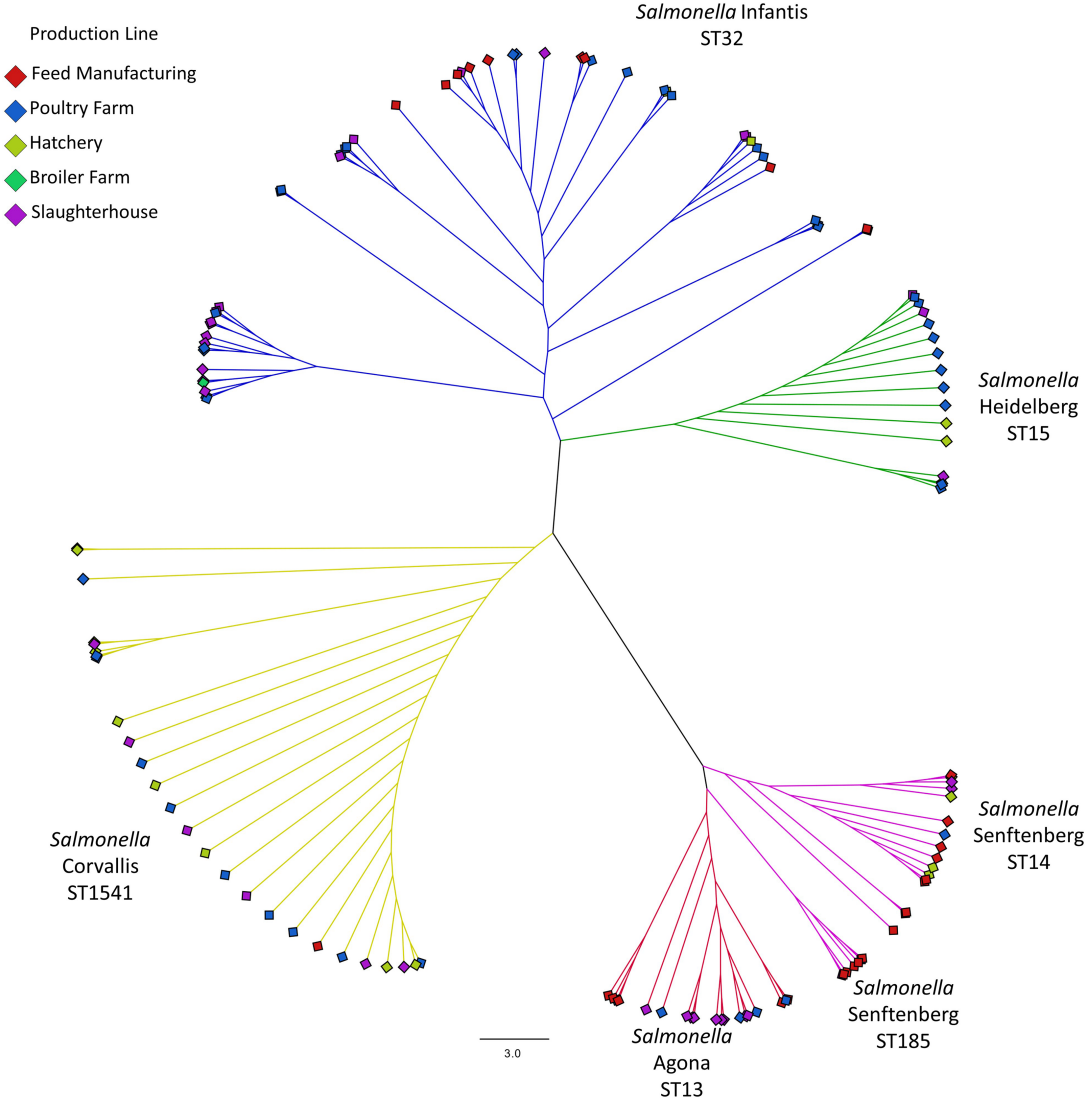
Phylogeny based on SNPs from the coregenome (546 genetic markers). The dotted lines demark the serotype group and their typification, while diamonds at the extremes of each branch correspond to the origin of the strain within the production line, coded by colors.

According to the phylogeny based on the multiple alignment for the core SNPs of 546 genetic determinants present in the 133 isolates (Figure 1), we found 6 clusters that corroborated the genome typification and their grouping by serotype. In addition, it is highlighted that the serotype Infantis shows clades with isolates present in several areas of the production line, while the Corvallis and Heidelberg serotypes have few differences between the isolates of the clade, suggesting that their contamination has a clonal origin and that these two strains had then spread throughout the production line. However, the presence of the Senftenberg ST185 clade is predominant in the manufacture of the food. A similar grouping is observed with the Agona serotype, where a clade group was isolated exclusively from the feed manufacture, separating itself from the rest of the production line.

**Figure 2:**
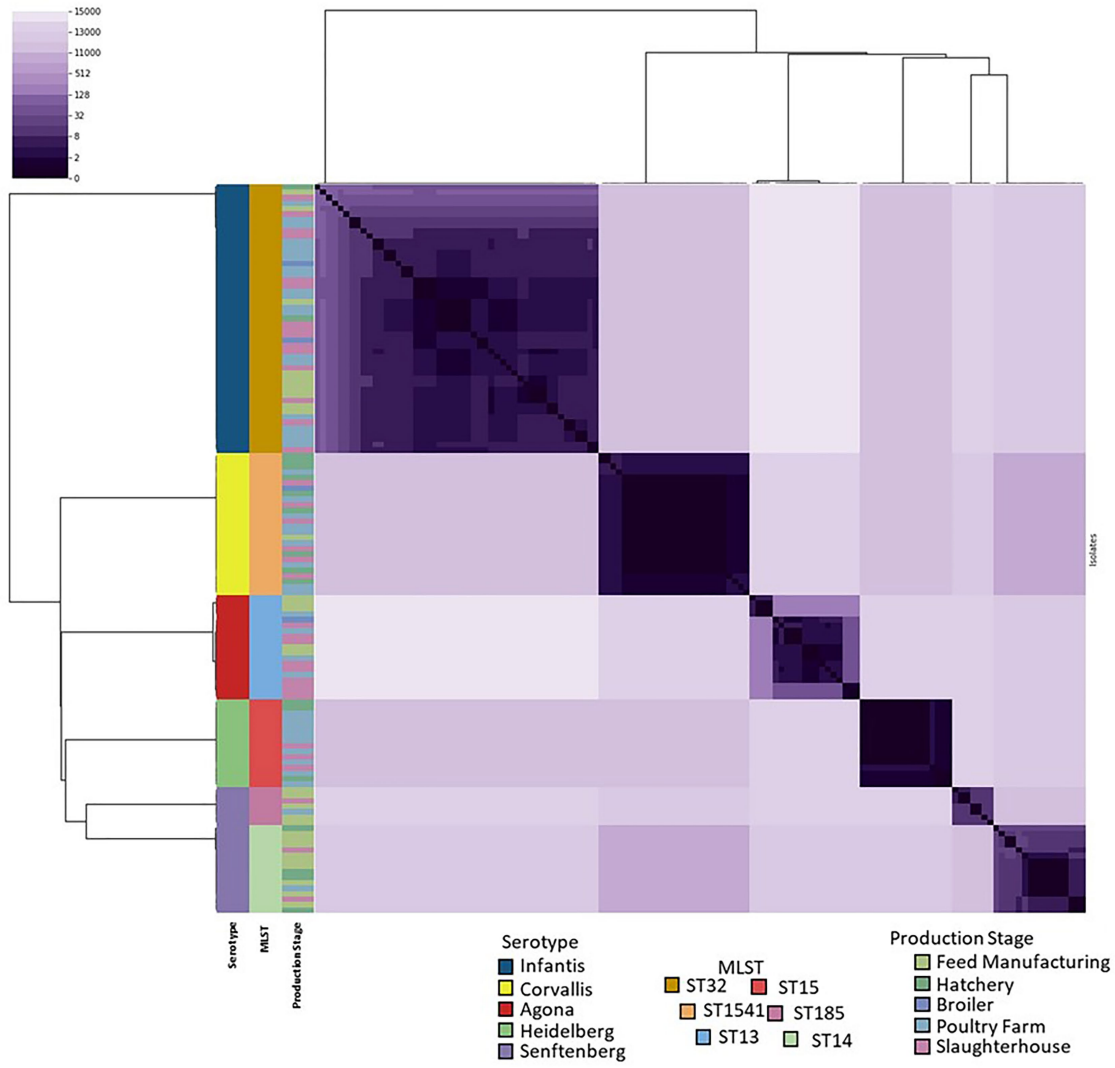
Heatmap of pairwise SNP distances of the 133 genomes. The dendrogram corresponds to the hierarchical clustering of the SNP distance matrix data. The colors on the left correspond to the serotype, genome typing and origin of the isolate within the production line.

The pairwise distance matrix between the genomes resulted in a range of 0–14,993 between all the isolates (Figure 2). The maximal distance found among the isolates of Agona serotypes was 199, and was lower in the others (Infantis 123, Heidelberg 2, Corvallis 4, and Senftenberg ST185 and ST14 12 and 31, respectively). However, among the Senftenberg ST strains, their distance ranges from 11,522 to 11,544, coinciding with the result of the presence of two different strains in the Senftenberg serotype.

### 3.2. Replicon detection for plasmids and mobile elements

We detected plasmid replicon-associated genes in at least 104 of the 133 isolates (Table 2). Moreover, our results indicate the coexistence of multiple replicons in 20 isolates, of which Col, Col3M, IncFIB and IncI1-Iα are the most commonly identified. We reconstructed 88 draft plasmid sequences from references corresponding to the identified replicons. We determined that 56 draft plasmid sequences possessed resistance elements, including the IncFIB plasmid present in serotypes Infantis, the IncI1-Iα plasmid found in Heidelberg, Senftenberg and Corvallis, as well as the Col plasmid present in various isolates. We identified insertion sequences and transposonic elements in the plasmid drafts. The most frequent insertion sequences were IS26, IS91, IS200, IS256, IS630, IS1326, IS903, ISEch12, ISEc57, ISVsa3, and ISEcp1. Furthermore, in the IncFIB plasmid of Salmonella Infantis, we identified sequences for attl recombination corresponding to class I and class II integrons. On the other hand, the IncI1-Iα plasmid also contained the attl insertion sequence only for class I integrons.

**Table 2:** Prediction of the presence of replicons from plasmids in *Salmonella* isolates.

Serotype	Replicon^*^	Total
Agona	Col(pHAD28)^**^	1
	Col3M	9
	IncH1B(pNDM-CIT)	2
	IncFIB(pHCM2)	1
Corvallis	Col(pHAD28)^**^	23
	Col3M	2
	IncI1-Iα^**^	1
Infantis	Col(pHAD28)^**^	3
	IncFIB(pN55391)^**^	47
	IncX4	2
Heidelberg	Col(pHAD28)^**^	15
	IncI1-Iα^**^	7
Senftenberg	Col(pHAD28)^**^	5
	IncH1B(pNDM-CIT)	5
	IncI1-Iα^**^	1
	IncFIB(K)	1
	IncFIB(S)	4

* Replicon detected by PlasmidFinder.

** Present in the reconstructed plasmid draft sequence (this work).

### 3.3. Virulence factors and virulence profiles

From Whole Genome Secuence (WGS) analysis, we identified a total of 29,931 virulence factors among all isolates. Of these, 51.3% (15,378/29931) correspond to adherence determinants and biofilm formation, and 42.3% (12,651/29931) to secretion system effectors, such as components of the type III secretion system including the pathogenicity islands 1 and 2, along with their respective effectors. The remaining virulence factors 6.4% (1901/29931) correspond to competitive advantages, colonization, virulence regulation and stress adaptation, iron, magnesium and phosphorus metabolism, toxins, and siderophores (for more information see Supplementary Table S2).

Overall, the identified virulence-associated genetic components are homogeneous among strains. For example, in relation to adherence and biofilm formation, we found fimbrial elements commonly distributed among the isolates, such as csg, fim, lpf, and non-fimbrial elements shdA and sinH. All isolates have the type III secretion systems of SPI-1 and SPI-2. On the other hand, Salmonella Heidelberg presents the highest frequency of sodC1, which has been associated with stress response. We highlight the main differences are found between isolates possessing IncFIB and IncI1-Iα plasmids. IncFIB carriers possess ccdAB and vapBC toxin-antitoxin systems for plasmid maintenance; in addition, IncFIB possesses a fae operon related to fimbrial formation, as well as a yersiniabactin operon related to siderophore formation. In contrast, IncI1-Iα plasmid carriers exhibit the toxin-antitoxin parAB systems for maintenance. It should be noted that in isolates carrying the IncI1-Iα plasmid, they also exhibit the colicin-IB toxin.

Antimicrobial resistant genes, and their relationship with mobile elements and their agreement with the resistance phenotype.

To further characterize the isolates, we used the CARD database to determine the existence of antimicrobial resistant genes in the obtained genomes (Table 3). There are 546 antimicrobial resistant genes, mostly assigned to the plasmid sequence drafts, except *fosA7*, present on the chromosome of the Heidelberg serotypes. The serotypes with the highest amount of antibiotic resistance genes were Infantis (447/546 resistance genes) remotely followed by Heidelberg (62/546 resistance genes).

**Table 3:** Antibiotic classes and resistant genes identified in *Salmonella* isolates.

Antimicrobial family or agent	Antimicrobial resistant gene	Serotype (N° MDR)					Agona (0)^*^	Corvallis (1)^*^	Heidelberg (7)^*^	Infantis (47)^*^	Senftenberg (1)^*^
Quinolone	*qnrB19*	1	22	17	1	5
Aminoglycosides	*aac(3)-IV*	0	0	0	42	0
	*ant(3′)-Ia*	0	0	6	47	1
	*aph(3′)-Ia*	0	0	0	31	0
	*aph(4)-Ia*	0	0	0	42	0
Beta-lactams	*bla*_CTX-M-65_	0	0	0	41	0
	*bla*_TEM-IB_	0	1	7	0	1
Trimethoprim	*dfrA1*	0	1	7	0	1
	*dfrA14*	0	0	0	36	0
Phenicol	*floR*	0	1	5	40	0
Fosfomycin	*fosA3*	0	0	0	19	0
	*fosA7*	0	0	16	0	0
Disinfectant	*qacEΔ1*	0	0	0	47	0
Sulfisoxazole	*sul1*	0	0	0	47	0
	*sul3*	0	0	6	0	1
Tetracycline	*tet(A)*	0	1	5	47	1

* Number of isolates that have multi-resistant profiles (>3 genes of antibiotic resistance).

In addition, we determined that there are susceptible genetic profiles, without the presence of identified resistance genes. In this context, all Senftenberg ST185 and 11 Senftenberg ST14 isolates, the majority of Agona isolates (18 of 19 isolates identified) and 9 Heidelberg isolates are susceptible to antibiotics. All the other isolates analyzed show resistance and multi-resistant profiles, the latter reflecting the presence of possible mobile elements.

Multi-resistant profiles with the highest diversity of antibiotic resistant genes are present in strains belonging to the serotype Infantis (Table 3), including 12 genes in a single isolate. In addition, *tet(A)*, *sul1*, *ant(3′)-*Ia and *qacEΔ1* genes, which represent resistance to tetracycline, sulfonamides, aminoglycoside and quaternary ammonium disinfectant, respectively, are present in all Infantis isolates with an MDR profile.

Overall, 56 of the 133 isolates have MDR profiles, with *Salmonella* Infantis isolates accounting for 87.9% of them (47 of 56 MDR). From the results drawn from the resistance profiles of these isolates, we can infer that the poultry farm itself harbors the greatest amount of MDR profiles of all the sites sampled in this study. In contrast, the lowest frequency of isolates with an MDR profile is found in the feed manufacturing stage (Supplementary Table S1).

**Figure 3:**
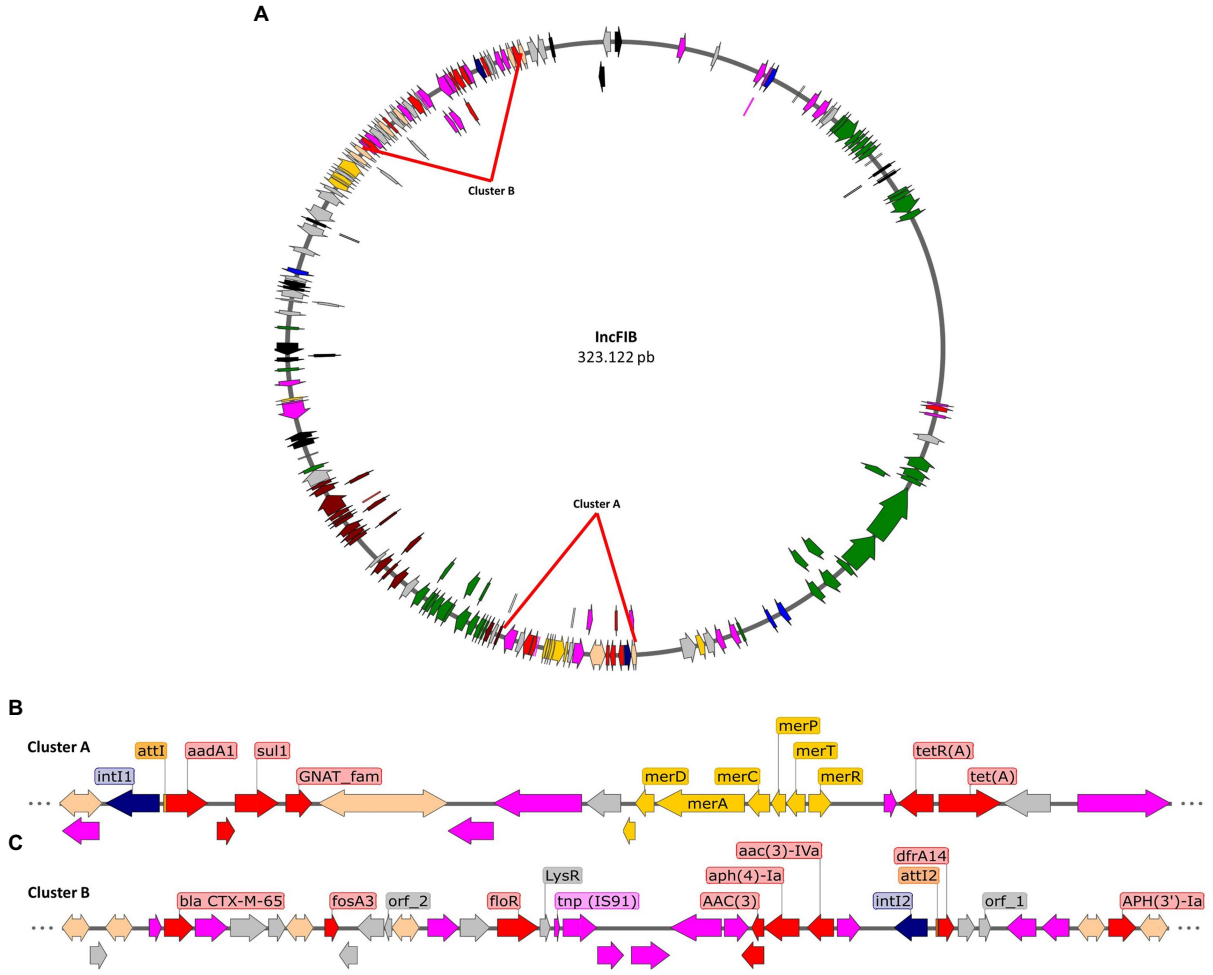
Representation of the IncFIB plasmid draft of serotype Infantis isolates. **(A)** Draft of the IncFIB plasmid, highlighting the position of clusters A and B that contain diverse mobile elements that include antibiotic resistance genes. **(B)** Genomic context of mobile and resistance elements in cluster A. **(C)** Genomic context of mobile and resistance elements in cluster B. Transposons in fuchsia arrows, integrases in blue arrows, sequence of integration in orange rectangles, antibiotic resistance genes in red arrows, metal resistance genes in yellow arrows, virulence genes in green arrows, conjugation genes in brown arrows, and other passenger genes in gray arrows.

Furthermore, we aimed to describe the genomic context of resistance genes to relate them to mobile elements identified in the draft plasmids from the *Salmonella* isolates. For instance, in the IncFIB plasmid draft (Figure 3A), two clusters with high densities of mobile elements and resistance genes were found. Cluster A, of approximately 21 kbp (Figure 3B), has a Tn2 family transposon that contains the *tetR* and *tetA* genes that confer resistance to tetracyclines and includes a class 1 integron together with a *mer* operon for mercury resistance. The integron has insertions of the antibiotic resistance genes *ant(3′)-Ia*, *qacEΔ1* and *sul1*, which confer resistance to aminoglycosides, quaternary ammonium and sulfonamides, respectively. This cluster is suggested to be well conserved as it is present in all Infantis isolates containing the IncFIB plasmid. On the other hand, cluster B (Figure 3C), is 32 kbp in size, and is made up of various mobile elements, mainly of the IS*26* type. These mobile elements carry aminoglycoside resistance genes, such as *aph(3′)-Ia*, *acc(3)-IVa*, *aph(4)-Ia*, as well as fosfomycin resistance genes, *fosA3*. On the other hand, the presence of a transposon that contains the *floR* gene, for amphenicol resistance, is characterized by having two passenger genes that correspond to *virD2* and a gene from the *lysR* transcriptional regulator family. This cluster also includes a transposon that is truncated by the insertion of another mobile element, which contains the *fosA3* gene. This transposon includes the *bla*_CTX-M-65_ gene, an extended-spectrum beta-lactamase, together with the *yncD-Cter*/*ΔiroN* gene, involved in the formation of siderophores.

**Figure 4:**
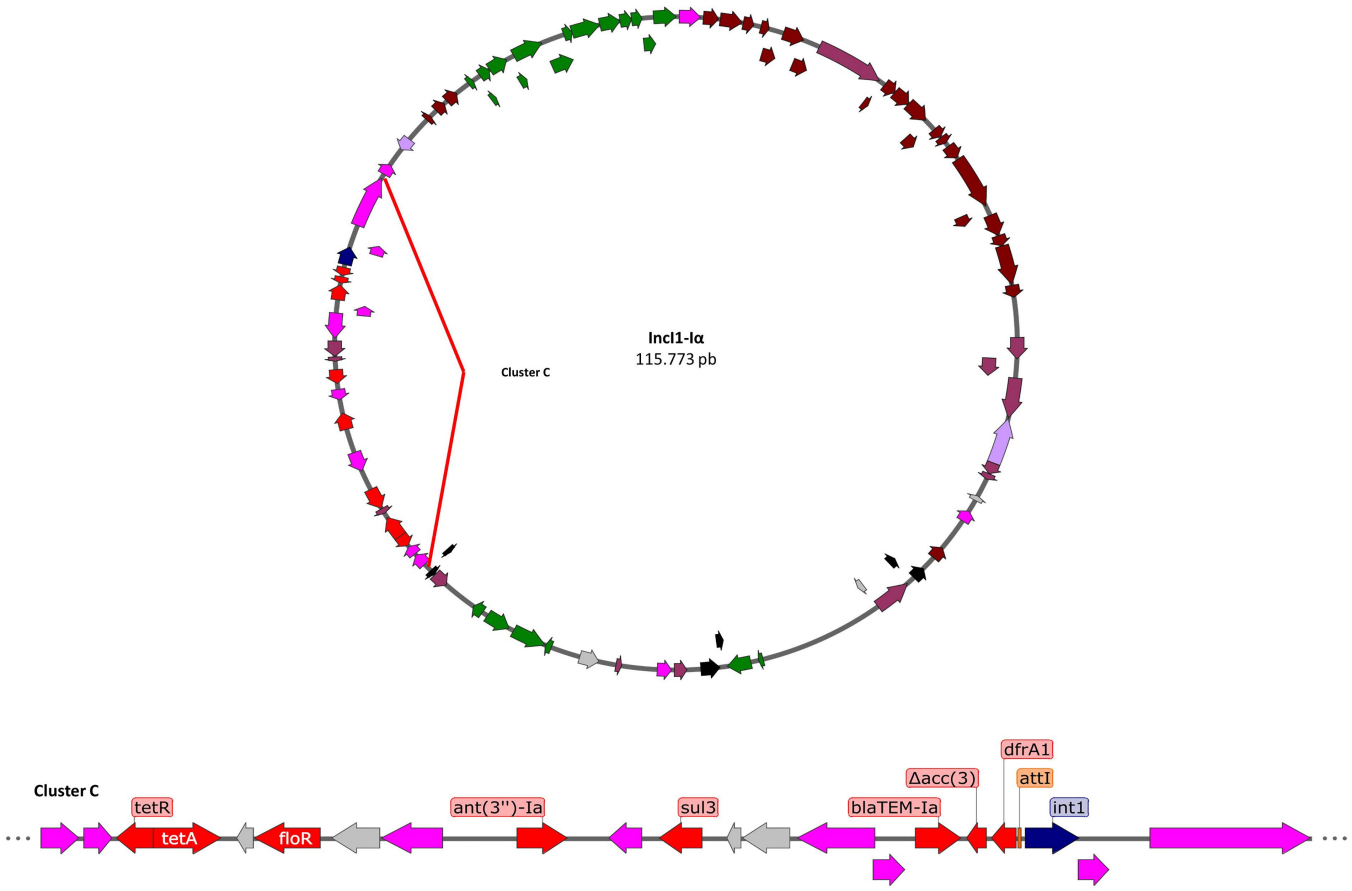
Genomic contexts of the resistant genes (red arrows) in plasmid IncI1-Iα. Transposons in fuchsia arrows, integrases in blue arrows, sequence of integration in orange rectangles, antibiotic resistance genes in red arrows, metal resistance genes in yellow arrows, virulence genes in green arrows, conjugation genes in brown arrows, and other passenger genes in gray arrows.

In addition, all carriers of the IncI1-Iα plasmid (Figure 4) possess the *bla*_TEM-Ib_ and *dfrA1*, encoding an extended-spectrum beta-lactamase, and trimethoprim resistance, respectively. The *dfrA1* gene belongs to a class I integron that also has a pseudogenized *aac(3)-IV* gene, suggesting that the insertion of the transposon containing the *bla*_TEM-Ib_ gene disrupts the *aac(3)-IV* sequence. On the other hand, the IncI1-Iα plasmid has the *sul3* gene present in an IS*26* transposon along with two other reading frames. On the other hand, the *ant(3′)-Ia* gene is found between two transposons, IS*26* and IS*Vsa3*, and is also adjacent to *attC* insertion sequences, suggesting that this gene belonged to an integron. Other identified resistance genes, *floR* and *tetA*, are found together in a transposon along with passenger genes such as *lysR*.

The drafts suggest that there are variants in the structure of the plasmid that contain the mobile elements and their resistance genes, identifying a cluster (hot spot) of approximately 17 kb where the resistance genes are concentrated.

Finally, we evaluated the consistency of the genotype and phenotype of resistance to the corresponding antibiotic (Table 4). In this context, the percentage agreement and Cohen’s kappa coefficient were evaluated for each antibiotic. We observed a range of values for kappa between 0.687 and 1.0, with the lowest values recorded for beta-lactams and trimethoprim/sulfamethoxazole. Ceftriaxone resistance is due to the presence of extended spectrum beta-lactamase genes such as *bla*_CTX-M-65_ and *bla*_TEM-1b_, while sulfamethoxazole/trimethoprim is associated with *drfA* and *sul* genes. Therefore, the visible differences at the phenotypic level could be linked to the variability of the mobile elements within the plasmids of these isolates.

**Table 4:** Agreement between genotypes and phenotypes of *Salmonella* strains.

Antimicrobial family or agent	Genotype for resistant phenotype^*^		Genotype for susceptible phenotype		Agreement (%)	Kappa	Resistant	Susceptible	Resistant	Susceptible
**Aminoglycosides**						
Gentamicin	37	5	1	90	95.49	0.893
Kanamycin	31	0	3	82	97.41	0.936
Streptomycin	54	0	0	78	100	1.00
**Beta-lactams**						
Penicillin						
Ampicillin	44	5	8	76	90.23	0.793
**Cephalosporins**						
Cefazolin	36	13	1	83	89.47	0.762
Ceftriaxone	38	11	8	76	85.71	0.689
**Fosfomycin**						
Fosfomycin	34	1	0	97	99.24	0.98
**Phenicol**						
Chloramphenicol	43	2	7	81	93.23	0.853
**Tetracyclines**						
Tetracycline	48	5	4	76	93.23	0.858
Trimethoprim/sulfamethoxazole	33	10	8	82	86.47	0.687

* Genes identified in CARD database.

## 4. Discussion

This investigation aimed to study and compare the structure and genetic dynamics of the *S. enterica* isolates obtained from the production line in a poultry farm. We detected the presence of the Infantis, Heidelberg, Agona, Corvallis, and Senftenberg serotypes in several stages of production (Table 1), of which *Salmonella* Infantis was the serotype with greatest prevalence in this study. This is in accordance with the evidence that proposes that this is an emerging serotype of concern worldwide (EFSA, 2019; Lapierre et al., 2020; Li et al., 2020; Mejía et al., 2020; Pardo-Esté et al., 2021). On the other hand, typification of the genome determined the presence of six strains, where the presence of *Salmonella* Infantis ST32 is of special interest, as it has been previously-described that this is a multi-resistant, emergent strain that carries the plasmid type pESI incFIB containing several resistant elements (Alba et al., 2020; Kürekci et al., 2021; Bertani et al., 2022). In addition, we detected the presence of the serotype *Salmonella* Heidelberg ST15, which is known to be a carrier of the IncI1-α plasmid, a plasmid associated with the *bla*_TEM-1b_ gene, an extended-spectrum beta-lactamase (Castellanos et al., 2018; van den Berg et al., 2019). The phylogeny results suggest that there is no correlation between the stage of the production line and the grouping of the strains, for all the serotypes evaluated (Figure 1), as concluded previously by Pardo-Esté et al. (2021) for *Salmonella* Infantis isolates. In addition, the phylogeny and the study of the distance by pairs of the SNPs confirmed that the diversity and propagation of the strains along the production line is independent of the serotype, denoting a high genetic relationship and a low variability in the core genome (Figure 2), suggesting that propagation is of clonal origin at all the sampled stations. This finding could be due to *Salmonella* contamination circulating and re-entering the industrial environment, as these bacteria has been linked to the process of poultry meat at various stages, including presence in incoming animals, in feed production, and even in personnel (Marin et al., 2022).

The differences between virulence factors are mainly associated with the difference between serotypes, with genomic mobile elements being the main factor contributing to the variability. Moreover, the presence of antimicrobial resistance genes also contributes to the pathogenicity of carrier bacteria. In this sense, the presence and increased expression of virulence genes linked to pathogenicity in bacteria with MDR profiles (García et al., 2011; Long et al., 2022; Salaheen et al., 2022) has been described. In this context, the presence of plasmids and other mobile elements in emerging pathogenic bacteria is very common, since the main route of acquisition of these elements is horizontal gene transfer (Vinayamohan et al., 2022).

Furthermore, in this study we found the IncFIB and Incl1-α plasmids in the strains (Figures 3 and 4), which contain a wide variety of resistance elements that have been described and linked to mobile genetic elements (Partridge et al., 2018). These plasmids have been reported in other serotypes, including IncFIB type pESI in Agona and Senftenberg, as well as in isolates that harbor both plasmids (Cohen et al., 2022; dos Santos et al., 2022), conferring a severe public health risk. However, there are some discrepancies (Table 4) between the genotype and phenotype of the strains, which could be attributed to several factors, such as promoter regions, secondary structures in the Shine-Dalgarno region, and the presence of unknown or undescribed mechanisms. (Davis et al., 2011). We found the greatest presence of resistant profiles in the Poultry Farm and Slaughterhouse stages, associated with the presence of mobile genetic elements, of which the Infantis and Heidelberg serotypes were particularly multi-resistant (>=7 resistant genes per isolate; Table 2). Both serotypes have *bla*_TEM-Ib_ and *bla*_CTX-M-65_ genes encoding extended-spectrum beta-lactamase present on plasmids, a recurrent trait in resistant bacteria in the poultry industry (Saliu et al., 2017). The presence of *bla*_TEM-Ib_ is common in plasmids; as such, Heidelberg serotypes with these characteristics have been described in chicken meat imported from the Netherlands (van den Berg et al., 2019). On the other hand, *bla*_CTX-M-65_ in Infantis has been described in the United States as a strain of clinical importance since it is directly associated with an emerging MDR strain (Tate et al., 2017; Brown et al., 2018).

The results highlight a progressive increase in resistance in the bacteria that remain in the production line, suggesting the existence of critical points of contamination. Research has previously described that these points are related to exsanguination and evisceration, as well as to cages of contingency and transport as the main reservoir (Marin et al., 2022), resulting in the release from the host of a high density of microorganisms of different origins, contaminating equipment, and personnel.

The intensive use of antibiotics, cleaning, and disinfection protocols in the poultry industry is another aspect that must be considered when analyzing the persistence of bacterial contamination along the production line. The constant and indiscriminate use of such compounds can select for tolerance and resistance in emerging bacterial strains (Mahnert et al., 2015). On the other hand, it has also been described that the ability to form a biofilm is an important trait that is related to pathogenicity and resistance to antimicrobials (Borges et al., 2018; Sun et al., 2019; Obe et al., 2021), as bacteria contained within the biofilm are 1,000 times more tolerant to antibiotics and disinfectants. Furthermore, the cooperation between different bacteria promotes mutual survival in an industrial setting (Dieltjens et al., 2020). In this context, biofilm formation has also been reported to promote the spread of mobile genetic elements (Madsen et al., 2012).

Additionally, the current public health situation associated with the SARS-CoV-2 pandemic has led to the widespread use of disinfectants that can contribute to the appearance of bacteria with MDR profiles, with the potential to harbor mobile genetic elements (Fuga et al., 2022). Therefore, it is important to conduct genomic surveillance and understand genetic dynamics in emerging bacteria such as *Salmonella* in an industrial setting.

## 5. Conclusion

Mobile genetic elements produce emerging bacteria with a high capacity for resistance to antimicrobials, constituting a danger to public health and a risk to food safety. The high genetic relationship between the bacteria of the poultry industry highlights contamination of the entire production chain by emerging bacteria. This should be considered a reservoir of MDR bacteria with the potential to be transmitted to humans, either directly or through poultry-derived products. Such transmission is facilitated thanks to mobile genetic elements.

## Data availability statement

The datasets presented in this study can be found in online repositories. The names of the repository/repositories and accession number(s) can be found at: https://www.ncbi.nlm.nih.gov/, PRJNA890630.

## Author contributions

GK, CP-E, JC-S, and CS: conceptualization, formal analysis, and visualization. GK: data curation. LA-T and CS: funding acquisition, resources. GK, CP-E, and CS: investigation. GK, PZ, JO-P, NG, MZ, and CS: methodology. CS: project administration. JO-P, MT, JV, and CS: supervision. GK and CS: validation. GK and CP-E: writing—original draft. GK, CP-E, JC-S, MT, and CS: writing—review and editing. All authors contributed to the article and approved the submitted version.

## Funding

This research was sponsored by ANID (Agencia Nacional de Investigación y Desarrollo de Chile) grants. CS was funded by ANID-FONDECYT Regular 1210633 and ECOS-ANID 170023. JC-S was funded by ANID 2021 Post-Doctoral FONDECYT 3210156. LA-T was funded by FONDECYT N° 1191019. CP-E was funded by ANID-FONDECYT Post-Doctoral 3230189.

## Conflict of interest

The authors declare that the research was conducted in the absence of any commercial or financial relationships that could be construed as a potential conflict of interest.

## Publisher’s note

All claims expressed in this article are solely those of the authors and do not necessarily represent those of their affiliated organizations, or those of the publisher, the editors and the reviewers. Any product that may be evaluated in this article, or claim that may be made by its manufacturer, is not guaranteed or endorsed by the publisher.
